# The evaluation of central-line–associated bloodstream infection (CLABSI) preventability at an academic institution

**DOI:** 10.1017/ash.2022.113

**Published:** 2022-05-16

**Authors:** Leon Hsueh, Daniel Uslan, Annabelle De St. Maurice

## Abstract

**Background:** In 2008, the hospital-acquired conditions (HACs) initiative labeled central-line–associated bloodstream infections (CLABSIs) as preventable “never events” that could no longer be reimbursed by Medicare. However, some patients have inherent unpreventable etiologies for bacteremia, such as obstructive biliary malignancies. We assessed the number of CLABSIs that were reasonably preventable. **Methods:** We examined all CLABSI cases at 2 academic medical centers over a 2-year period (2019–2021). We established 3 categories of CLABSIs: (1) preventable CLABSI (pCLABSI); (2) end-of-life CLABSI (EOL-CLABSI), which were CLABSIs that were caused by underlying disease processes in patients who were nearing the end of their lives due to a debilitating comorbidity; and (3) definition-based (dCLABSI), which met NHSN criteria for a CLABSI but, based on the pathogen and the clinical situation, likely occurred as a consequence of a patient’s comorbidities. Two experienced infectious diseases physicians (D.U. and A.S.M.) reviewed the charts of each patient with a CLABSI and, based on expert opinion, determined the category for each CLABSI. **Results:** Over the 2-year period, 147 CLABSIs were identified among the 2 hospitals, 66 (44.9%) of which occurred in an ICU. Most CLABSIs were pCLABSIs, making up 99 CLABSIs (67.3%). In comparison, 20 cases were categorized EOL-CLABSIs (13.6%), although 26 cases were dCLABSIs (17.7%), and 2 cases could not be classified. There was no difference in the distribution of CLABSI types in an ICU versus a non-ICU setting (χ^2^ *P* = .265). However, we detected microbiologic differences between pCLABSIs, EOL-CLABSIs, and dCLABSIs (χ^2^ *P* < .001), with gram-positive cocci making up the large majority of pCLABSIs (62.6%), followed by *Candida* spp (24.2%). Gram-negative bacilli (GNR) made up 11.1% of pCLABSIs. In comparison, GNRs were more prevalent in EOL-CLABSIs and dCLABSIs, making up 30.0% and 38.5% of each CLABSI type, respectively. **Conclusions:** Two-thirds of CLABSIs were deemed preventable. Central lines are important for managing critically ill patients, many of whom have inherent risk factors for bloodstream infections. EOL-CLABSIs highlight the potential for early care discussions to avoid CLABSIs at the end of a patient’s life and to avoid unnecessary blood cultures for patients on comfort care. Additionally, the pCLABSI distinction allows hospital epidemiology teams to focus on the CLABSI cases that can realistically be prevented with appropriate central-line care, techniques, and hand hygiene. Creating these categories allows hospital systems to use more targeted approaches for improving CLABSI rates.

**Funding:** None

**Disclosures:** None

